# Effects of Growth Factors on In Vitro Culture of Neonatal Piglet Testicular Tissue Fragments

**DOI:** 10.3390/cells12182234

**Published:** 2023-09-08

**Authors:** Fahar Ibtisham, Tat-Chuan Cham, Mohammad Amin Fayaz, Ali Honaramooz

**Affiliations:** Department of Veterinary Biomedical Sciences, Western College of Veterinary Medicine, University of Saskatchewan, Saskatoon, SK S7N 5B4, Canada; fmi065@mail.usask.ca (F.I.); tatchuan.cham@usask.ca (T.-C.C.); ma.fayaz@usask.ca (M.A.F.)

**Keywords:** in vitro spermatogenesis, male infertility, tissue culture, growth factors, porcine testicular tissues

## Abstract

In vitro spermatogenesis (IVS) has important applications including fertility preservation of prepubertal cancer patients; however, thus far, IVS has only been achieved using mouse models. To study the effects of growth factors on the maintenance of testicular tissue integrity, germ cell numbers, and potential induction of IVS using a porcine model, we cultured small testicular fragments (~2 mg) from 1-wk-old piglets under six different media conditions (DMEM + 10%KSR alone or supplemented with GDNF, bFGF, SCF, EGF, or a combination of all) for 8 weeks. Overall, tissues supplemented with GDNF and bFGF had the greatest seminiferous tubule integrity and least number of apoptotic cells. GDNF-supplemented tissues had the greatest number of gonocytes per tubule, followed by bFGF-supplemented tissues. There was evidence of gradual Sertoli cell maturation in all groups. Moreover, histological examination and the expression of c-KIT (a marker of differentiating spermatogonia and spermatocytes) and STRA8 (a marker of the pre/meiotic stage germ cells) confirmed the induction of IVS in all groups. However, GDNF- and bFGF-supplemented tissue cultures had greater numbers of seminiferous tubules with spermatocytes compared to other groups. In conclusion, overall, GDNF and bFGF supplementation better maintained the tissue integrity and gonocyte numbers and induced IVS in cultured testicular tissues.

## 1. Introduction

Male germline stem cells (MGSCs) commence as primordial germ cells (PGCs), emanating from the proximal epiblast during embryonic development. Once PGCs migrate to and colonize the genital ridge, they evolve into gonocytes. During neonatal development, gonocytes give rise to spermatogonial stem cells (SSCs), with their simultaneous potential for self-renewal (to maintain their population) and undergoing differentiation to produce sperm through the complex developmental process of spermatogenesis. As such, SSCs are the only adult stem cells contributing to the next generation. However, in domestic animals, due to the difficulty in unequivocal identification of SSCs, we have limited knowledge and means to use their full potential in assisted reproductive technologies and stem cell therapy [1]. Therefore, to unveil the great potential of SSCs for such applications, it is crucial to define optimal culture conditions for their long-term in vitro maintenance, propagation, and differentiation. The long-term culture of rodent SSCs is relatively well established [2], while this knowledge is largely lacking in domestic animals [3]. However, the short-term culture of SSCs has been reported in different farm animals, including pigs [4,5,6], goats [7,8,9,10], buffalo [11,12,13], and sheep [14].

The mammalian testis consists of two functionally and structurally separate sections, namely seminiferous tubules (STs) and interstitial compartment. While STs are home to Sertoli and germ cells as the site for spermatogenesis, the interstitial compartment contains Leydig and supporting cells as the site of androgen production. Moreover, STs are surrounded by peritubular myoid cells, which contract to accelerate the movement of sperm along the ST lumen toward the efferent ducts and epididymis [15]. SSCs reside at the stem cell niche, formed by various somatic cells, where Sertoli cells are considered to be the most important cells. Sertoli cells can be functionally classified as either immature (fetal/neonatal) or mature (adult). Immature Sertoli cells are involved in testis formation and regression of the Müllerian ducts via secretion of the anti-Müllerian hormone (AMH) during the fetal period. In contrast, immature Sertoli cells provide a microenvironment suitable for SSC self-renewal during the neonatal period. Furthermore, this microenvironment provides various signals including growth factors to regulate the self-renewal and differentiation of SSCs [16]. Approaching puberty, Sertoli cells undergo maturation, and their function switches to support spermatogenesis by producing growth factors crucial for germ cell differentiation. As Sertoli cells mature, they lose their proliferative ability, form inter-Sertoli cell tight junctions, and express several other functions not seen in immature Sertoli cells [17].

Glial cell line-derived neurotrophic factor (GDNF), released by Sertoli cells, helps regulate the proliferation and differentiation of SSCs [18]. GDNF is a member of the transforming growth factor-beta superfamily [19], which was first isolated based on its ability to support the development of embryonic midbrain dopaminergic neurons in vitro [20]. The expression of GDNF was then observed in different organs and tissues, including the intestine, kidney, muscle, cartilage, and lungs, suggesting that GDNF also has physiological roles in non-nervous systems [21,22]. The crucial role of GDNF in regulating SSC numbers was shown when mice with impaired GDNF signaling showed a gradual loss of SSCs, while its pan-ectopic overexpression promoted germ cell hyperplasia and ultimately tumor formations [6]. Subsequently, it was reported that supplementation of GDNF promotes short-term and/or long-term in vitro expansion of mouse SSCs [23,24]. GDNF has also been reported to increase the number of SSCs in buffalo [13] and goats [10] during in vitro culturing. Recently, we observed that GDNF increases porcine gonocyte/SSC proliferation in a dose-dependent manner in a two-dimensional (2D) cell culture [5]. It has been shown that GDNF promotes the proliferation of gonocytes/SSCs via activation of the Src kinase(s) and a PI3K/Akt pathway to increase *N-myc* gene expression [25]. Interestingly, in vivo experiments using mice have suggested that GDNF increases SSC self-renewal by blocking differentiation rather than actively promoting their proliferation [26]. Moreover, it is believed that GDNF favors self-renewal at the expense of differentiation by promoting the expression of *Numb* and degradation of the Notch pathway, which normally promotes the differentiation of SSCs [27].

Studies have also demonstrated that the basic fibroblast growth factor (bFGF) regulates GDNF production in Sertoli cells [28]. Adding bFGF in combination with GDNF increased the long-term self-renewal of mouse and hamster SSCs [24,29], while bFGF alone was ineffective in producing a similar result [24,29]. However, we recently reported that bFGF alone or combined with GDNF could increase the number of porcine gonocytes/SSCs in a culture. Interestingly, after 28 days of gonocyte culturing, groups treated with bFGF alone or in combination with GDNF did not differ, suggesting that bFGF alone is also sufficient for the long-term culture of porcine gonocytes [5]. 

Epidermal growth factor (EGF) receptor signaling is important for cell growth, proliferation, survival, and differentiation [30]. In testes, Leydig cells are the major source of EGF; however, germ cells also start producing EGF at the onset of spermatogenesis. The production of EGF from Leydig cells during early germ cell development appears to help in SSC proliferation, while the production of EGF from germ cells is associated with the onset of meiosis [31]. A recent study showed that adding 10 ng/mL of EGF in culture media increases the number of porcine SSC colonies. EGF facilitates SSC proliferation in an endocrine manner through its receptors on Sertoli cells including ErbB1, ErbB2, and ErbB4 [32]. Moreover, the mature form of EGF has been found in spermatocytes, spermatids, and Sertoli cells in mouse testes [33]. These findings suggest that EGF has a crucial role in the proliferation and differentiation of germ cells. Similarly, stem cell factor (SCF) has also been shown to play a crucial role in normal spermatogenesis. In testes, SCF is produced by Sertoli cells [34] and interacts with the c-Kit receptor on Leydig cells [35,36]. SCF interaction with the c-Kit is also important for PGC migration and survival [37,38]. Moreover, SCF is crucial for spermatogonial maintenance, proliferation, and adhesion [39]. Importantly, we have shown that implantation of testis cell aggregates from various donor species under the back skin of recipient mice results in de novo formation (regeneration) of testis tissue. Using this novel testis regeneration model, we observed that even a brief pre-implantation exposure of neonatal testis cells to GDNF, EGF [40], bFGF, or SCF can have long-term effects by altering the development of testicular STs and/or rete-testis [41].

Studies on the maintenance and proliferation of gonocytes/SSCs are vital in understanding their biology and identifying growth factors that are crucial for their self-renewal and differentiation. Therefore, several studies, using 2D in vitro culture conditions, have aimed to identify key growth factors required for gonocyte/SSC proliferation. However, culturing testicular tissue fragments is advantageous compared to isolated cells (in 2D conditions), because tissue fragments maintain the necessary 3D testis microenvironment required for both testis development and spermatogenesis. Therefore, the present study was designed to provide a first indication of the effects of different key growth factors (GDNF, bFGF, EGF, and SCF, alone or in a combination) on in vitro maintenance of tissue integrity, gonocyte/SSC numbers, and potential induction of IVS in cultured neonatal porcine testicular tissue fragments using the organ culture model (gas–liquid interface).

## 2. Materials and Methods

### 2.1. Study Design

**Figure 1:**
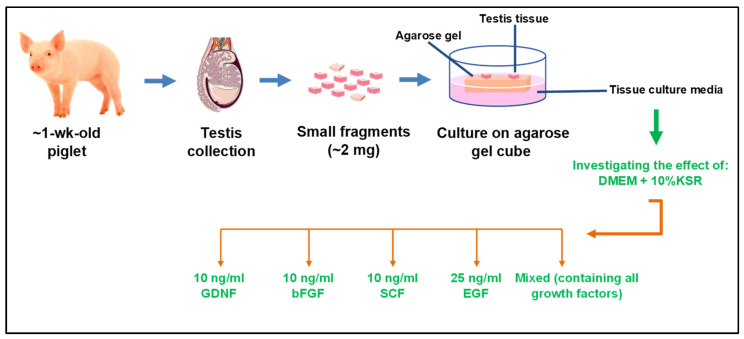
Schematic representation of the experimental design. Testes from neonatal donor piglets were collected and divided into small (~2 mg) testicular fragments. Four fragments from each group were placed on an agarose gel cube soaked in the designated media. All fragments from each well (in 6-well plates) were collected as a sample per time point. Samples were taken every 2 weeks for 8 weeks and analyzed using TUNEL, immunofluorescence, and H&E to examine the tissue integrity, germ cell numbers, germ cell differentiation stage, and number of dead cells. H&E: Hematoxylin and eosin stain; TUNEL: Terminal deoxynucleotidyl transferase (TdT) dUTP nick-end labeling.

The present study was designed to examine the in vitro effects of various growth factors on piglet testicular tissue fragments. The concentrations of growth factors used in the present study were based on our previous in vivo and in vitro studies on pig testis cells and tissue [5,40,41], or relevant other work [7]. As schematically shown in Figure 1, testes collected from 1-wk-old piglets were divided into small testicular fragments and cultured in 6 differently supplemented media for 8 weeks where samples were taken every 2 weeks. This was to optimize the culture media composition for maintaining tissue integrity, germ cell population, and induction of IVS. The entire experiment was replicated four times. We had hypothesized that differently supplemented media would affect (1) the long-term integrity of testicular tissue fragments in the culture, (2) the relative number of germ cells in these fragments, and (3) the induction of germ cell differentiation.

### 2.2. Testis Collection and Preparation

Testes were collected from neonatal (~1-wk-old) Yorkshire-cross piglets (Camborough-22 × Line 65; PIC Canada, Winnipeg, MB, Canada) through aseptic castration at a university-affiliated swine center. Collected testes were transported to the lab and prepared using previously reported protocols with minor modifications [6]. Briefly, the testis parenchyma was cut into small fragments of ~2 mg, following the removal of tunica albuginea, rete-testis, and excess connective tissue. 

### 2.3. Culture of Tissue Fragments

Testicular tissue fragments were cultured on top of 1.5% agarose gel cubes soaked in Dulbecco’s modified Eagle medium (DMEM; catalogue no. 10-013-CM; Mediatech) + 10% knockout serum replacement (KSR; catalogue no. 10828028; Thermo Fisher Scientific, Carlsbad, CA, USA), and 1% *w/v* antibiotics along with individual and/or combined concentrations of GDNF, bFGF, SCF, and/or EGF (Table 1) for 8 weeks. The control group had testicular tissue fragments cultured in basic media (DMEM + 10% KSR) without any growth factors [6]. During the 8 weeks of study, culture media were changed every 2–3 days, and the cultures were maintained under standard culture conditions (at 37 °C in 5% CO_2_) in a humidified incubator. 

### 2.4. Histological Examination of Cultured Testicular Tissue Fragments

Every 2 weeks, random samples were collected and fixed in Bouin’s solution (catalogue no. 1120-31; Richa Chemical Company, Pocomoke City, MD, USA) for 8–10 h, followed by rinsing with 70% ethanol and processing in an automated tissue processor (Leica ASP300S, Leica Biosystems, Buffalo Grove, IL, USA). Processed tissues were then paraffin-embedded and sectioned at a thickness of 5 µm, and for histological assessment, the largest sections were used for H&E staining.

A semi-quantitative analysis in a blinded manner was conducted to evaluate the integrity of cultured testicular tissue fragments. Briefly, STs (*n* = 256 per time point per group) of the testicular tissue fragments were scored based on 4 parameters: cellular cohesion, adhesion of cells to the basement membrane, easy distinction of germ cells and Sertoli cells, and proportion of pyknotic nuclei. The scores ranged from 4 to 1, where score 4 corresponded to the best ST integrity and score 1 to the worst integrity. In addition, the average number of gonocytes per ST was quantified by calculating the number of gonocytes in round or almost round tubule cross sections (*n* = 122 per time point per group) in 5 randomly selected fields per slide. ST diameters were measured in the round or almost round tubule cross sections (*n* = 122 per time point per group) using ImageJ software (version 1.53e).

### 2.5. Quantification of Apoptotic Cells

A terminal deoxynucleotidyl transferase biotin-dUTP nick-end labelling kit (TUNEL Assay Kit—HRP-DAB-ab206386, Abcam Inc, Toronto, ON, Canada) was used according to the supplier’s instructions to identify apoptotic cells. The total number of positively stained apoptotic cells per sample cross-section (*n* = 4 per time point per group) was counted and the counting was cross-validated with ImageJ.

### 2.6. Immunofluorescence (IF) and Immunohistochemistry (IHC) 

Following deparaffinization and dehydration, the 5 µm thick tissue sections, prepared as described above, underwent antigen retrieval. For antigen retrieval, slides were soaked in a boiling citrate buffer (pH = 6.3; catalogue no. H-3300; Vector Lab, Burlington, ON, Canada) for 20 min in the microwave. Afterwards, the samples were left at room temperature for 30 min to allow for cooling down. Subsequently, the samples underwent three rounds of rinsing with DPBS and were then treated with 5% BSA for 20 min at room temperature to avoid non-specific bindings. The samples were then incubated with the respective primary antibody, which included AMH (1:100 *v*/*v*; catalogue no. NBP2-43670, Novus, Littleton, CO, USA), AR (1:100 *v*/*v*; catalogue no. MBS9614407, MyBioSource, San Diego, CA, USA), c-KIT (1:100 *v*/*v*; catalogue no. MBS9614162, MyBioSource), STRA8 (1:100 *v*/*v*; catalogue no. MBS9611993, MyBioSource), or CYP17A1 (1:50 *v*/*v*; catalogue no. SC-374244; Santa Cruz Biotechnology, Santa Cruz, CA, USA), for 1 h in a humidified chamber at room temperature. This was followed by overnight incubation at 4 °C. For IF, this was subsequently followed by three rounds of rinsing with DPBS and a 1 h incubation with a secondary antibody (mouse anti-rabbit IgG, catalogue no. sc-2359, Santa Cruz Biotechnology) in a humidified chamber at room temperature. Finally, after rinsing with DPBS, the sections were incubated with a mounting media containing DAPI (catalogue no. H-1500-10, Vector Labs) for 2–3 min followed by examination using fluorescent microscopy. For IHC, the incubation of 1 h with the secondary antibody (ImmPRESS reagent peroxidase-universal anti-mouse/rabbit, catalogue no. MP7500, Vector labs) was performed at room temperature in a humidified chamber. The sections were then rinsed with DPBS and incubated with DAB (ImmPACT DAB, catalogue no. SK4105, Vector lab) for 5–10 min and counterstained with hematoxylin for 5 min. The sections were subsequently mounted using mounting media (Sigma-Aldrich, catalogue no. 03989, Oakville, ON, Canada).

### 2.7. Analysis of IF and IHC Images 

In the present study, most of the data were extracted using histological examination of H&E slides. However, the H&E findings were further cross-validated using specific antibodies, including verification of differentiating germ cells in the ST cross-sections (*n* = 256 per group per time point) using c-KIT and STRA8. 

The expression of AMH and AR was analyzed using ImageJ. Briefly, images were converted to grayscale and positively stained areas were segregated using thresholding to quantify the relative percentage of areas exhibiting positive staining. 

### 2.8. Statistical Analysis

The data are mean values along with the standard error of the mean (SEM). A significance level of *p* < 0.05 was deemed significant. To analyze the morphometric data of ST cross-sections, a three-way analysis of variance (ANOVA) was used followed by Tukey’s HSD post hoc test, for determining the pairwise differences. The number of apoptotic cells per cross-section, ST cross-section diameters, number of gonocytes per ST, and number of differentiating germ cells were analyzed using a two-way ANOVA (the group and time as main factors) followed by Tukey’s HSD as the post hoc test. To analyze the expression data of AMH and AR at different time points (week 2 and 8), 2 independent sample T-tests were performed. The Statistical Package for Social Sciences (IBM SPSS Statistics, version 25.0, IBM Inc., Armonk, NY, USA) was used to conduct all analyses. 

## 3. Results

### 3.1. Tubular Integrity of Testicular Tissue Fragments during Culture

**Figure 2:**
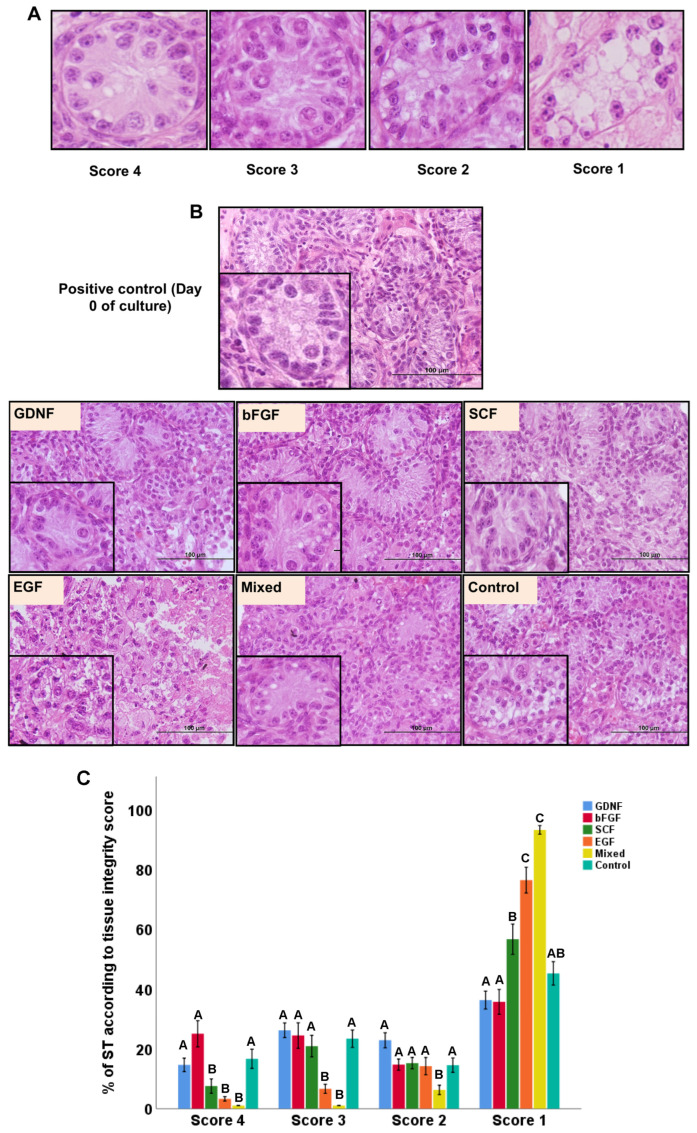
Tissue integrity of cultured testicular fragments during culture. (**A**) Representative histological photomicrographs of seminiferous tubules (STs) according to their integrity scores where score 4 corresponds to the best ST integrity and score 1 denotes the worst integrity. (**B**) Histological representation of testicular tissue fragments in different media at week 4 of culture. Overall, morphologically, GDNF-, bFGF-, and SCF-supplemented groups better maintained the integrity of STs. (**C**) Data showing the integrity scoring of STs in different culture media. Among growth-factor-supplemented groups, GDNF- and bFGF-supplemented groups maintained the highest percentage of well-preserved (score 4) and lowest percentage of worst score category of STs (score 1). Data with different letters in different growth factor groups or different time points within each score category differ significantly (*p* < 0.05). Scale bar: 100 μm.

**Figure 3:**
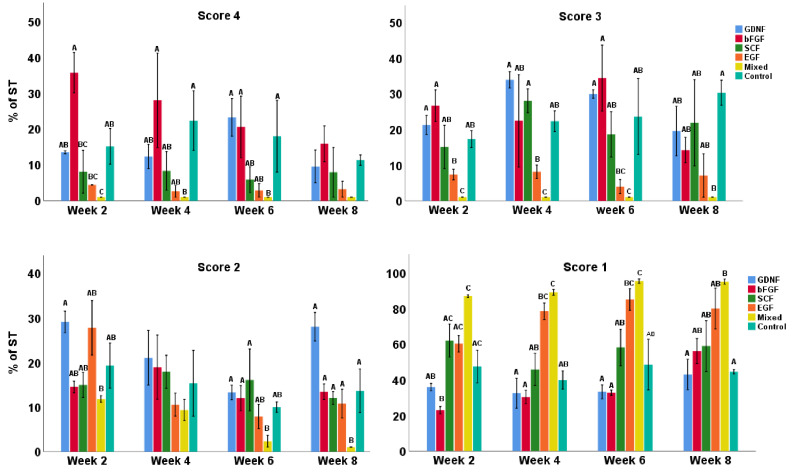
Data showing the integrity scoring of STs over the culture period in different growth-factor-supplemented groups. Data with different letters in different growth factor groups or different time points within each score category differ significantly (*p* < 0.05).

Seminiferous tubule cross-sections were scored in terms of integrity (from 4 to 1, i.e., best to worse morphology), as shown in representative histological sections in Figure 2A. In terms of overall morphology, GDNF-, bFGF-, and SCF-supplemented media better maintained the integrity of STs (Figure 2B). When comparing the tissue integrity scores, a significant interaction was found between media and the week; therefore, the data were split based on the score type and reanalyzed. Both media and the week of the culture significantly affected testicular tissue integrity (*p* < 0.05). Control, GDNF-, and bFGF-supplemented groups had significantly (*p* < 0.05) greater numbers of well-preserved (score 4) STs compared to SCF, EGF, and mixed groups. For score 3 (intermediately preserved integrity), the control, GDNF-, bFGF-, and SCF-supplemented groups had significantly (*p* < 0.05) greater numbers of STs compared to EGF and mixed groups. For score 2 (low intermediately preserved integrity), the EGF group had a significantly (*p* < 0.05) lower number of STs compared to all other groups. In the category of worst-score STs (score 1), GDNF- and bFGF-supplemented groups had significantly (*p* < 0.05) lower numbers of STs compared to SCF, EGF, and mixed groups. In contrast, the control group had a significantly (*p* < 0.05) lower number of worst-category STs (score 1) than only EGF and mixed groups (Figure 2C). Moreover, over the culture period, overall, the control, GDNF-, and bFGF-supplemented groups were better at maintaining the integrity (score 4 and 3) of STs (Figure 3). 

### 3.2. Cell Apoptosis during Culture of Testicular Tissue Fragments

**Figure 4:**
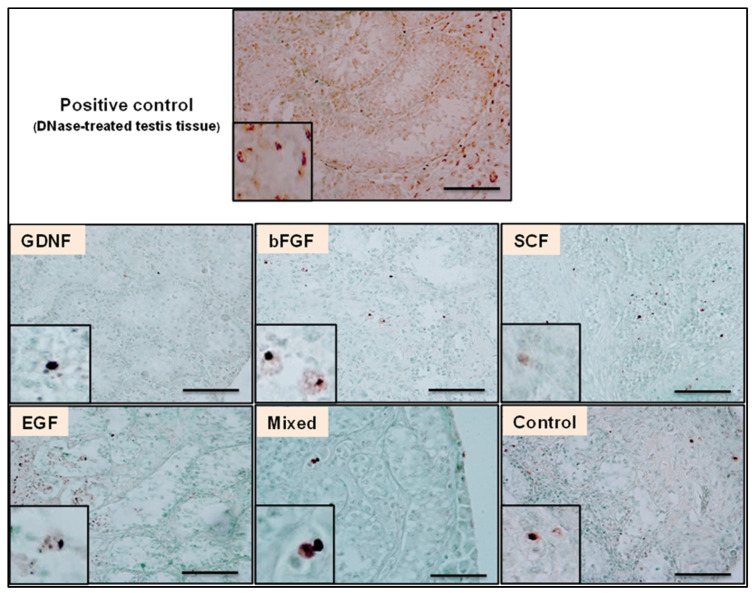
Representative photomicrographs of TUNEL-stained cells in different culture media at week 4 of culture. Scale bar: 100 µm.

**Figure 5:**
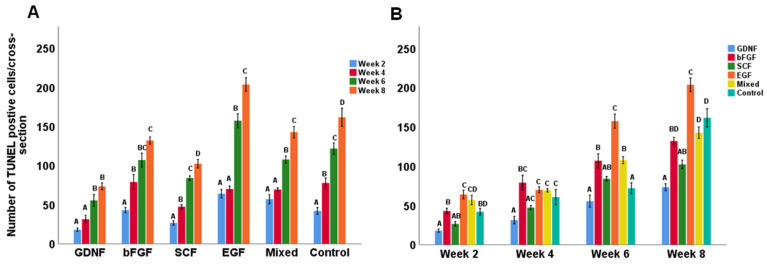
Number of TUNEL-positive cells in cultured testicular tissue fragments. (**A**) Data showing the number of TUNEL-positive cells per cross-section in different media over the period of culture. (**B**) Data showing the number of TUNEL-positive cells per cross-section over the period of culture in different culture media. Data with different letters within each media supplement group differ significantly (*p* < 0.05).

Apoptotic cells were identified using the TUNEL assay. Figure 4 shows representative photomicrographs of TUNEL-positive cells observed in different media groups. When comparing the number of TUNEL-positive cells, an interaction was found between media and the week; therefore, the data were split based on the week and reanalyzed. For all groups, the culture period significantly affected the number of TUNEL-positive cells per cross-section (*p* < 0.05). Although the number of TUNEL-positive cells increased over time in all groups (Figure 5A), overall, the GDNF-supplemented group better maintained the viability of cultured testicular cells compared to other groups (Figure 5B).

### 3.3. Seminiferous Tubule Diameters (μm) and Gonocyte Populations during Culture

**Figure 6:**
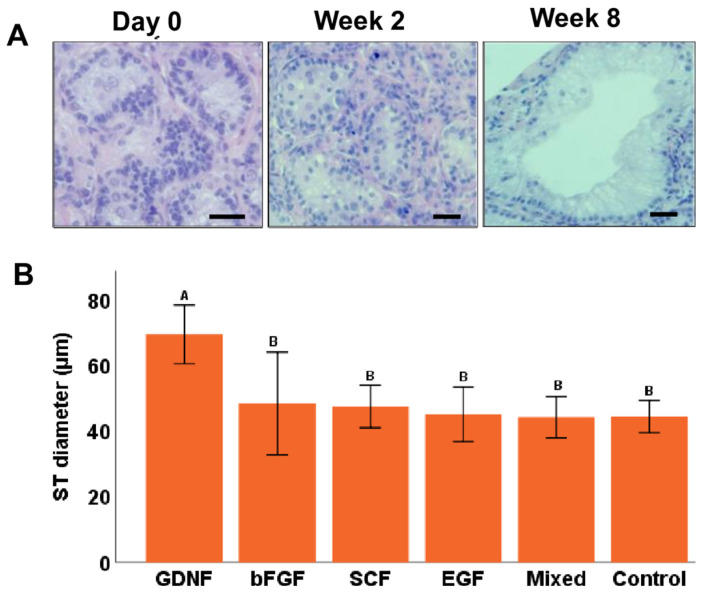
Diameter of seminiferous tubules (STs) in testicular tissue fragments during culture. (**A**) Representative photomicrographs of ST of testicular tissue fragments cultured in GDNF-supplemented media. (**B**) Diameter of STs in testicular tissue fragments in different groups at week 8 of culture. The GDNF-supplemented group had a significantly higher ST diameter compared to other groups. Groups with different letters differ significantly (*p* < 0.05). Scale bars: 20 µm.

At day 0, average ST diameters were 33.4 ± 0.76 µm, and during in vitro culturing, these diameters increased (Figure 6A). When comparing the diameters of STs during culturing, an interaction was found between media and the week; therefore, the data were split based on the week and reanalyzed. Only at week 8 of culturing was a significant (*p* < 0.05) difference observed among different media groups, where the GDNF-supplemented group had the greatest diameter of STs compared to other groups (Figure 6B).

**Figure 7:**
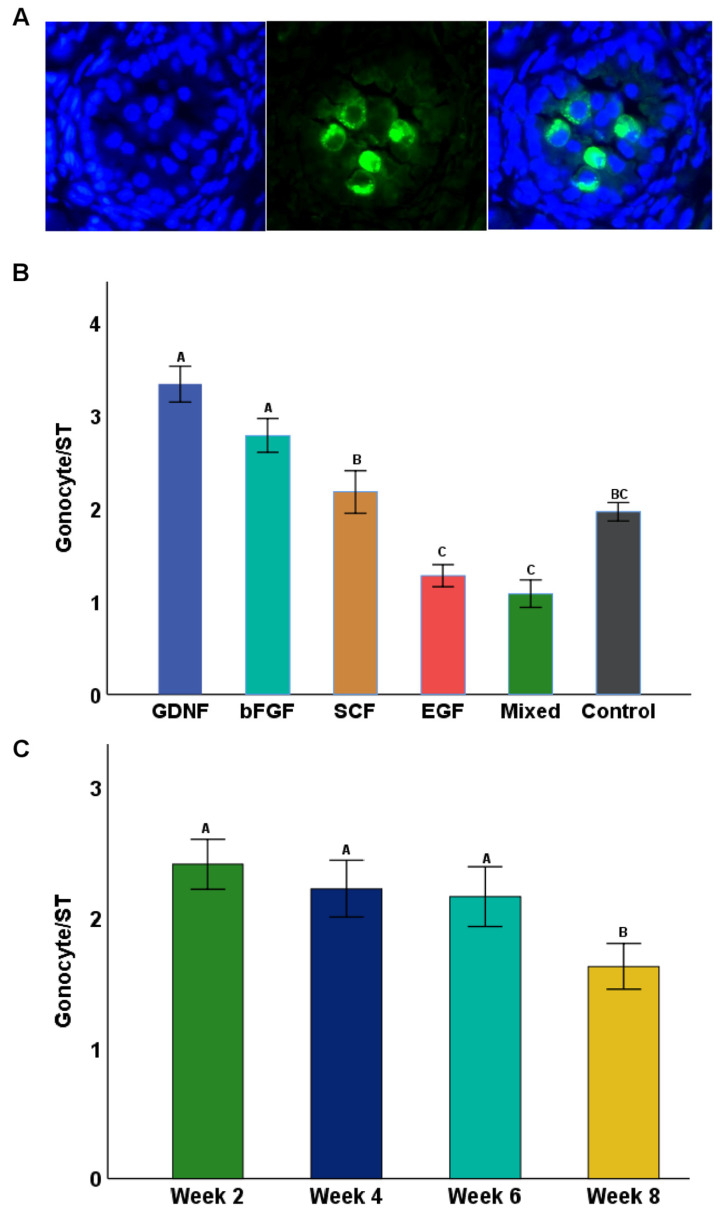
Immunostaining identification of gonocytes in cultured testicular tissue fragments. (**A**) Representative photomicrographs of gonocytes (immature germ cells) present in testicular tissue fragments cultured in GDNF-supplemented media (week 4) using immunostaining with Dolichos biflorus agglutin (DBA). (**B**) Data showing the number of gonocytes per seminiferous tubule (ST) cross-section in different culture media. GDNF- and bFGF-supplemented groups had significantly higher numbers of gonocytes compared to others. (**C**) Data showing the number of gonocytes per ST cross-section over the period of culture. The number of gonocytes decreased significantly at week 8 of culture. Groups with different letters differ significantly (*p* < 0.05). Scale bars: 20 µm.

Figure 7A shows the Dolichos biflorus agglutin (DBA) confirmation of gonocytes within cultured testicular tissue fragments. At day 0, STs had 4.6 ± 0.34 gonocytes per ST. In cultured tissues, GDNF- and bFGF-supplemented groups had significantly higher numbers (*p* < 0.05) of gonocytes per ST cross-section than all other groups (Figure 7B). The number of gonocytes per ST cross-section decreased over the course of culturing; however, this was significant only at week 8 (Figure 7C).

### 3.4. Maturation of Sertoli Cells

**Figure 8:**
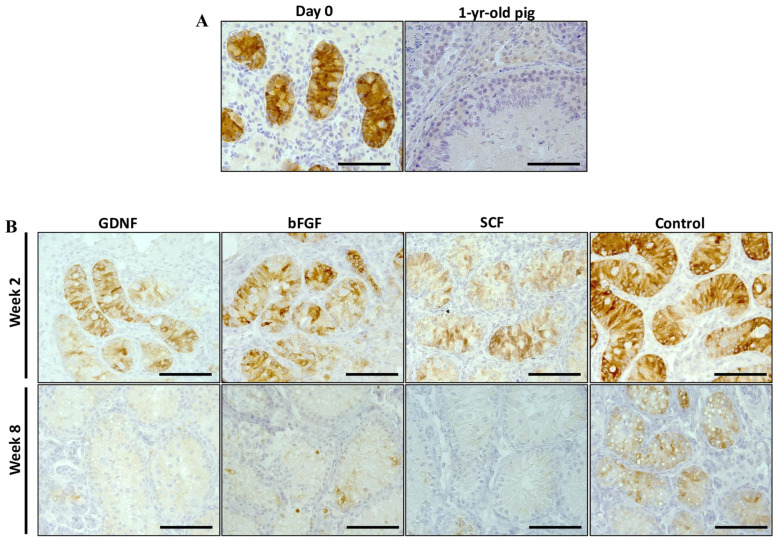
Immunohistochemistry examination of anti-Müllerian hormone (AMH) in seminiferous tubules (STs) of testicular tissue fragments during culture. (**A**) Testicular tissue samples of day 0 and mature pig served as positive controls for the expression of AMH. (**B**) AMH expression was assessed as a marker of immature Sertoli cells. At week 2, STs of the control group appeared to have strong to moderate staining. In contrast, most of the STs of the growth-factor-supplemented groups had moderate to low expression. At week 8, STs of all groups showed low to no expression of AMH. Scale bars: 100 µm.

**Figure 9:**
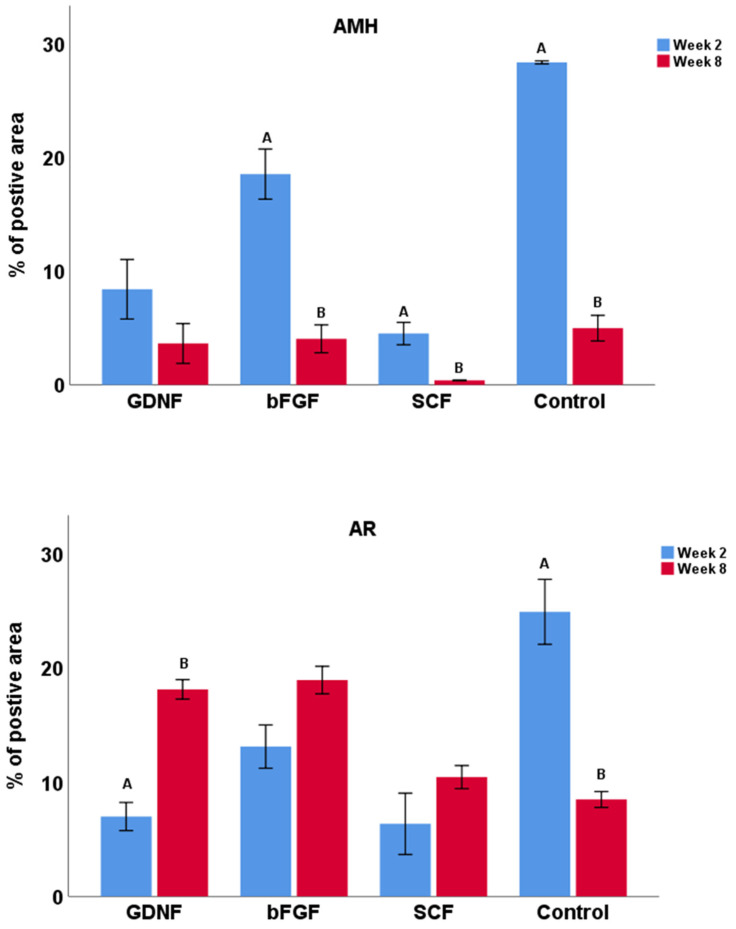
Percentage of AMH- and AR-positive stained area at week 2 and 8 of culture. In growth factor-supplemented groups, the expression of AMH decreased while the expression of AR increased over the period of culture. However, in the control group, expression of both AMH and AR decreased significantly over the period of culture. Data with different letters within each group differ significantly (*p* < 0.05).

Samples from weeks 2 and 8 of the best performing groups (GDNF, bFGF, SCF, and control) were selected to examine the maturational stage of Sertoli cells using the expression analysis of AMH (a marker for immature Sertoli cells) and androgen receptors (AR; a marker for mature Sertoli cells). For the AMH and AR expressions, we used a previously reported method of quantifying the relative percentage of positively stained areas per section [42,43]. Testicular tissue fragments of neonatal piglets on the first day of experiments (day 0) and a 1-year-old mature pig were used as positive controls (Figure 8A). The expression intensity of AMH decreased over the period of culturing in all groups (Figure 8B). The percentage of AMH positively stained areas decreased significantly (*p* < 0.05) over the culture period in bFGF, SCF, and control groups (Figure 9). 

**Figure 10:**
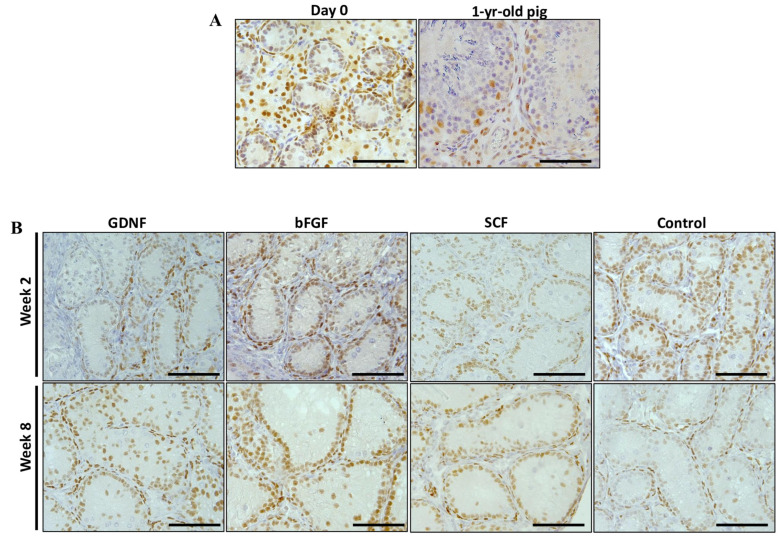
Immunohistochemistry examination of androgen receptors (AR) in seminiferous tubules (STs) of testicular tissue fragments during culture. (**A**) Testicular tissue samples of day 0 and mature pig served as positive controls for the expression of AR. (**B**) At week 2, Sertoli cells of control and bFGF groups appeared to have a stronger to moderate expression of AR. In contrast, GDNF and SCF groups had moderate to low expression. At week 8, Sertoli cells of all growth-factor-supplemented groups showed strong staining, and the control group showed low staining. Scale bars: 100 µm.

For the expression of AR, testicular tissue fragments of neonatal piglets on the first day of experiments (day 0) and a 1-year-old mature pig were used as positive controls. Leydig and peritubular myoid cells of day 0 testicular tissue fragments showed strong AR expression and minimal expression was observed in Sertoli cells. In contrast, Sertoli cells of testicular tissue fragments collected from a sexually mature pig showed strong positive expression (Figure 10A). 

The expression of AR increased over the period of culturing in all growth-factor-supplemented groups (Figure 10B). Although, in all growth-factor-supplemented groups, the percentage of AR positive areas increased over the period of culturing, and it differed significantly (*p* < 0.05) only in the GDNF-supplemented group. Interestingly, in contrast to all the latter mentioned groups, the expression of AR in the no-growth-factor control group decreased significantly (*p* < 0.05) over the period of culturing (Figure 9).

**Figure 11:**
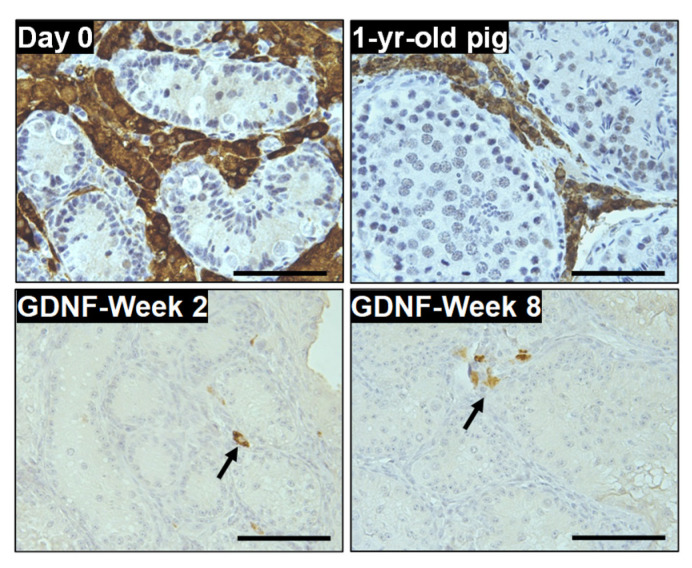
Immunohistochemistry detection of Leydig cells with CYP17A1 in GDNF-supplemented group at week 2 and 8 of culture. CYP17A1-positive cells were observed at both weeks 2 and 8 of culture (arrows). Testicular tissue samples of day 0 and mature pigs served as positive controls for the expression of CYP17A1. Scale bars: 100 µm.

Next, we investigated the expression of CYP17A1 in Leydig cells of the GDNF-supplemented (best-performing) group. CYP17A1-positive cells were observed at both week 2 and 8 of culturing (Figure 11).

### 3.5. Differentiation of Germ Cells during Culture of Testicular Tissue Fragments

**Figure 12:**
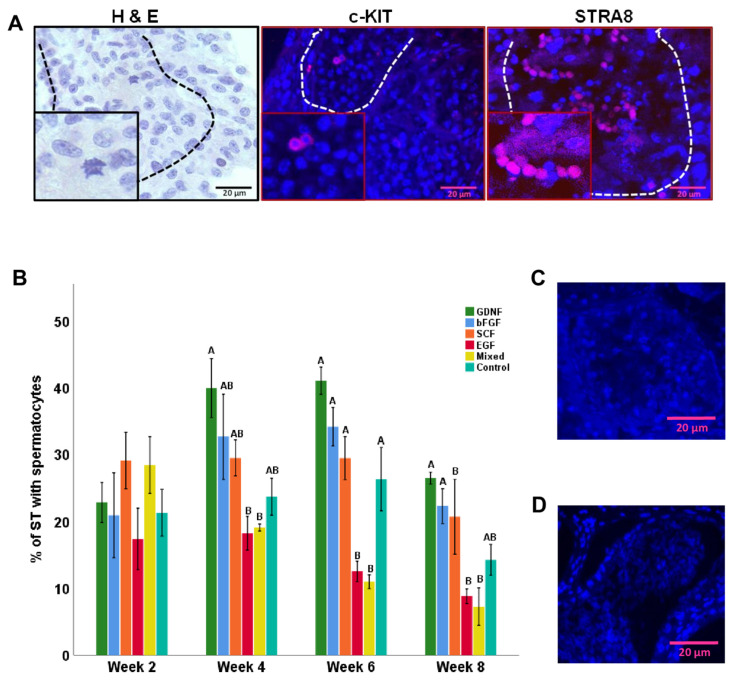
Immunofluorescence examination of differentiating germ cells (spermatogonia/spermatocytes) during culture. (**A**) Representative photomicrographs of differentiating germ cells in testicular tissue fragments cultured in GDNF-supplemented media (week 8) identified using hematoxylin and eosin (H&E) and the expression of c-KIT (a marker of differentiating spermatogonia and spermatocytes) or STRA8 (a pre-meiotic- and meiotic-stage-specific marker). (**B**) Data showing the percentage of seminiferous tubules (ST) with spermatocytes over the period of culture. (**C**,**D**) Negative controls for c-KIT and STRA8 expressions, respectively. Groups with different letters within each week differ significantly (*p* < 0.05). Scale bars: 20 µm.

Histological examinations confirmed the presence of differentiating germ cells in cultured testicular tissue fragments. These findings were further cross-validated with the immunofluorescence expression of c-KIT (a marker of differentiating spermatogonia and spermatocytes) and STRA8 (a pre-meiotic- and meiotic-stage-specific marker; Figure 12A). The percentage of STs containing spermatocytes were then analyzed. At weeks 4, 6, and 8 of culturing, numerically, the GDNF-supplemented group had the highest percentage of STs with spermatocytes, but it only differed significantly (*p* < 0.05) from EGF and mixed groups (Figure 12B). At week 8 of culturing, STs that were positive for spermatocytes in the GDNF-supplemented group had an average of 2.6 ± 0.15 spermatocytes. 

## 4. Discussion

We recently reported that with optimization of physical attributes (i.e., fragments’ size, preparation method, and serum source), neonatal piglet testicular tissue fragments can be maintained in vitro for up to 4 weeks. However, the number of gonocytes decreased over the period of culturing, and we did not observe any germ cell maturation [6]. In the present study, we set out to examine the effects of adding various growth factors to the media on maintaining tissue integrity and the maturational stage of germ cells and Sertoli cells. Here, we demonstrated that longer-term maintenance (to 8 weeks) and maturation of neonatal piglet testicular tissue fragments could be effectively achieved in vitro using a standard organ culture technique. To our knowledge, this is the first comprehensive assessment of the effects of key growth factors on the in vitro culture and maturation of porcine testicular tissue fragments.

Significant progress through 2D culture systems of IVS has allowed us [44] and other groups to achieve spermatogenesis using rodent models; however, evidence suggests that culturing intact testicular tissue fragments is a more robust approach for achieving significant IVS results [45]. Nonetheless, rodent models of IVS are of limited applicability in studying human spermatogenesis, largely because rodents have a very short prepubertal period (~21 days in mice vs. several years in humans). On the other hand, pigs may serve as an ideal animal model to study human spermatogenesis because of their anatomical and physiological similarities to humans and the relatively long prepubertal period (~6 months). Furthermore, routine castration of week-old piglets at swine facilities provides an easily accessible source of fresh testicular tissue and cells for various studies targeting testis function [16]. Therefore, some research groups have tried using 2D and organ culture models to achieve IVS using miniature pigs [46,47]. 

In the present study, we aimed to maintain testicular tissue fragments and induce IVS using the domestic pig model (longer puberty age compared to miniature pigs). In achieving IVS using neonatal piglet testicular tissue fragments, our first objective was to maintain tissue integrity during long-term culturing. Although we did not observe a significant difference among control, GDNF-, and bFGF-supplemented groups in maintaining the percentage of well-preserved (score 4) STs during culturing, the bFGF-supplemented group had the numerically highest percentage. It has been shown that bFGF can stimulate both DNA synthesis and cell multiplication of Sertoli [48] and Leydig cells [49] and their functions that play a critical role in the maintenance of STs [50,51]. Therefore, we speculate that bFGF supplementation maintained the ST integrity by increasing the activity of testicular somatic cells. Comparing the ST diameters, we observed that ST diameters increased over the period of culturing; however, they were significant only at week 8 of culturing. At week 8, the GDNF-supplemented group had STs with significantly higher diameters. We propose that the observed effects of GDNF on ST integrity and diameter are exerted through its promotion of Sertoli cell proliferation and tubulogenesis. This is because it has been shown that GDNF promotes the proliferation of Sertoli cells [22]. Previously, we also observed a similar effect of GDNF on tubulogenesis during in vivo implantation of porcine testis cells [40]. However, further work is needed to confirm the mechanism of the GDNF effect on testicular tubulogenesis. Although EGF had also been reported as being an important growth factor in maintaining testicular cells [32], in the present study, it showed no positive effects on maintaining the porcine testicular tissue fragments, the reasons for which need further work. 

As programmed cell death, testicular apoptosis is crucial for controlling the number of and eliminating defective germ cells during testicular development and spermatogenesis. However, during in vitro conditions, insufficient nutrient diffusion and/or poor gas exchange to cultured testicular tissue fragments could also lead to apoptosis by stimulating the production of reactive oxygen species [44]. In the present study, although, in all groups, the number of TUNEL-positive cells increased over the culture period, GDNF- and SCF-supplemented groups tended to better maintain the viability of testicular tissues, especially at later stages of the culture. Comparable protective effects of the latter growth factors were also observed in neural cells, the dental mesenchyme, human embryonic dopaminergic neurons, and testicular cells [52,53,54,55,56]. It has been reported that these exogenous growth factors protect cell death by activating the mitogen-activated protein kinase (MAPK) signaling pathway [57,58,59]. Various studies have shown that EGF acts as a survival factor that prevents apoptosis [60]. In the present study, we observed a significant increase in the number of TUNEL-positive cells in the EGF-supplemented group over the culture period. Our findings contradict a previous in vivo study in cryptorchid rats [61]. EGF dose differences could account for the discrepancy in results; in the in vivo study, 10 ng/mL was injected into the STs of the cryptorchid rat testis, while in the present study, 25 ng/mL was used for in vitro culturing of testicular tissue fragments. Furthermore, we used the TUNEL assay to detect cells with DNA breaks. However, recently, many studies have reported that TUNEL staining is not specific to only apoptotic cells as it labels all free 3′-hydroxyl termini regardless of the molecular mechanism that has led to their development [62,63,64]. Therefore, using a specific marker of apoptosis will further strengthen our observations. 

Various parameters, including Sertoli cell maturation, increased ST diameter, and the presence of post-meiotic germ cells, can be used to document the initiation of spermatogenesis [17]. The increase in the diameter of ST over the period of culturing was the first indication that cultured testis tissue was undergoing in vitro maturation. Next, we tracked the maturation of the Sertoli cell with the expression of AMH and AR. AMH is one of the earliest cell-specific proteins produced by Sertoli cells, which starts to be expressed at the 8th week of gestation in the human or 12.5 days post-coitum in the mouse [65,66]. The expression of AMH in Sertoli cells is then continuously observed during the fetal and postnatal periods, and up until the onset of puberty [67]. During the pubertal period, the expression of AMH starts to progressively decrease, which coincides with the formation of the blood–testis barrier and the onset of germ cell meiosis [68,69,70]. Overall, the expression intensity of AMH in STs decreased for all examined groups (from 2 to 8 weeks), indicating gradual maturation of Sertoli cells; however, among growth-factor-supplemented groups, bFGF- and SCF-supplemented groups had a significantly lower percentage of AMH-positive areas. In contrast, the expression of AR increased in all growth factor-supplemented groups (GDNF, bFGF, and SCF), compared to the control group. The increased expression of AR in Sertoli cells further validated our initial findings that Sertoli cells matured during in vitro culturing. Higher expression of AR in Sertoli cells of the control group at week 2 and lower expression at week 8 need further investigation to explore the reasons for the fluctuation. Regarding in vivo, the expression of AMH ends at day 110 [71] and the expression of AR in Sertoli cells is first observed at ~day 84 in pigs [72]. The presence of matured Sertoli cells at as early as 2 weeks of culturing suggests that our developed culture conditions accelerated the maturation of Sertoli cells. Furthermore, the expression of AR is regulated by testosterone and its derivative dihydrotestosterone (DHT), the binding of which begins the nuclear translocation and transcriptional regulatory function of AR [73]. Moreover, the production of testosterone is regulated by the gonadotrophin luteinizing hormone (LH) [74]. In the present study, strong expression of AR at week 8 of culturing also suggests that a certain degree of functional cell maturation of Leydig cells was achieved in vitro. Measuring testosterone levels in the culture media is required to further support our speculation. However, previously, using a novel neonatal porcine organoid model, we showed that neonatal pig Leydig cells indeed mature during in vitro culturing [75]. 

Gonocytes are the only type of germ cells present in the neonatal pig testes; therefore, maintaining their population during in vitro culturing of testicular tissue fragments is crucial in achieving IVS. In the present study, we also examined the individual and combined effects of GDNF, bFGF, SCF, and EGF on porcine gonocyte numbers and differentiation. GDNF-supplemented media had the highest number of early germ cells (gonocytes/spermatogonia) over the period of culturing, suggesting that GDNF has an important role in the maintenance of porcine early germ cells. This is in accordance with our recent findings using a 2D cell culture showing that GDNF increased the number of early germ cells in pigs [5], as it has for cells from mice [24,76], bovine [77], and humans [78]. GDNF appears to increase the number of early germ cells by activating various signaling cascades that trigger mitotic divisions in gonocytes/spermatogonia/SSCs [22,25]. While mice heterozygous for *Gdnf* are fertile, their number of in vivo STs with spermatogonia decrease with age [19]. In contrast, overexpression of *Gdnf* resulted in an accumulation of undifferentiated spermatogonia in STs [79]. In the present study, the bFGF-supplemented group was in second place in having the highest numbers of early germ cells over the period of culturing. These findings suggest that bFGF also has a direct or indirect role in the mitogenic and proliferative activity of germ cells and/or somatic cells [80]. Similar to our findings, bFGF supplementation increased the number of early germ cells in mice [24], bovine [78], pigs [5], and humans [81]. EGF has also been described as a spermatogonial stimulating growth factor [82]; however, in the present study, we observed that the EGF-supplemented group had the lowest number of early germ cells. Our findings contradict a previous report where EGF supplementation increased the number of porcine and buffalo SSC colonies [13,32]. The contrast in findings could also be due to the EGF dose differences and cell culture techniques; in the latter reports, a 10 ng/mL dose was used in a 2D cell culture, while in the present study, we used 25 ng/mL of EGF in a testicular tissue culture. Therefore, further investigation is warranted to find the optimal dose of EGF for piglet testicular tissue culturing. However, our findings are consistent with a previous report on bovine spermatogonia [77]. The number of early germ cells only decreased at week 8 of culturing, suggesting that overall, our developed culture technique was able to maintain germ cell numbers over the period of culturing.

KSR supplementation was deemed important in inducing complete spermatogenesis using immature mouse testicular tissue fragments [83,84,85]; however, since the actual composition of KSR is unknown, it is hard to make conclusions about the potential effects of various ingredients in the culture media. However, irrespective of medium composition, these organ/tissue culture models presented a proof-of-principle that complete IVS can be achieved using neonatal mouse donors [83,84,85]. Similarly, organ/tissue culture models have also shown promising results in achieving IVS in mini pigs and humans [43,47,86]. Therefore, in the present study, we also used the organ culture model to maintain and initiate differentiation of testicular tissue fragments of neonatal piglets. We confirmed the differentiation of germ cells in cultured testicular tissue fragments using H&E and expression of c-KIT and STRA8. The expression of c-KIT has been linked to the commitment of SSCs to differentiation [87], while the expression of STRA8 is crucial for the induction of meiosis, and hence it is usually expressed in pre-meiotic- and meiotic-stage germ cells [88]. In the present study, we observed that cultured testicular tissue fragments underwent maturation and produced differentiating germ cells. The number of STs containing differentiating germ cells increased over the period of culturing. Among the growth factor groups, GDNF-, bFGF-, and SCF-supplemented groups tended to have a high percentage of STs with spermatocytes. GDNF and bFGF have been reported to induce the initial steps of germ cell differentiation by upregulating the genes specific to germ cell differentiation in mice and humans, respectively [18,89]. Similarly, it has been suggested that SCF also plays a fundamental role in regulating the differentiation of SSCs [90]. A recent study showed that SCF improves in vitro differentiation of rat SSCs through upregulating the expression of *Prtm1*, *Stra8*, *c-Kit*, *Piwil2*, and *Oct4* genes [90]. Therefore, we speculate that these growth factors (GDNF, bFGF, and SCF) may have increased spermatocytes by upregulating the genes specific to germ cell differentiation. Furthermore, the maturation of Sertoli cells in the latter mentioned growth-factor-supplemented media further optimized the conditions to induce differentiation of germ cells. 

The presence of spermatocytes in pig testes in situ is first observed at day 110, followed by round spermatids at day 130, and spermatozoa at day 150 [71]. However, in the present study, we started observing the presence of spermatocytes as early as week 2 of culturing, suggesting that our culture conditions not only supported germ cell survival but also their accelerated differentiation. The lack of advanced-stage differentiated germ cells (i.e., spermatids or sperm) highlights the need for additional investigations into in vitro tissue culture requirements, including studies on effective concentrations of growth factors and their potential interactions, hormones (i.e., FSH, LH, or testosterone), and retinoic acid (RA). Indeed, the lack of advanced-stage differentiated germ cells in cultured testicular tissue fragments could be due to the absence of these or yet-to-be-discovered media components.

## 5. Conclusions

We demonstrated that in vitro maturation of neonatal piglet testicular tissue fragments can be induced using an optimized culture system. Supplemented growth factors caused various effects, including better maintenance of germ cell populations during culturing and accelerated Sertoli and germ cell maturation. These findings have important implications in expanding our ability to keep non-rodent testicular tissues in a culture for potential induction of complete in vitro spermatogenesis.

## Figures and Tables

**Table 1:** Culture media composition used to culture testicular tissue fragments.

Groups	Culture Media
1	DMEM + 10% KSR + 10 ng/mL GDNF
2	DMEM + 10% KSR + 10 ng/mL bFGF
3	DMEM + 10% KSR + 10 ng/mL SCF
4	DMEM + 10% KSR + 25 ng/mL EGF
5	DMEM + 10% KSR + 10 ng/mL GDNF + 10 ng/mL bFGF + 10 ng/mL SCF + 25 ng/mL EGF
6 (Control)	DMEM + 10% KSR (No growth factors)

## Data Availability

If additional data related to this study are required, please consult the corresponding authors.
